# New Insights into First-Line Therapy in Diffuse Large B-Cell Lymphoma: Are We Improving Outcomes?

**DOI:** 10.3390/jcm13071929

**Published:** 2024-03-27

**Authors:** Pau Abrisqueta

**Affiliations:** 1 Department of Hematology, Vall d’Hebron Hospital Universitari, Experimental Hematology, Vall d’Hebron Institute of Oncology (VHIO), Vall d’Hebron Barcelona Hospital Campus, Passeig Vall d’Hebron 119-129, 08035 Barcelona, Spain; pabrisqueta@vhio.net; 2 Departament de Medicina, Universitat Autònoma de Barcelona, 08193 Bellaterra, Spain

**Keywords:** diffuse large B-cell lymphoma, first-line therapy, novel therapies

## Abstract

Diffuse large B-cell lymphoma (DLBCL) is the most prevalent subtype of lymphoma, comprising heterogeneous patient subgroups with distinctive biological and clinical characteristics. The R-CHOP combination (rituximab, cyclophosphamide, doxorubicin, vincristine, and prednisone) has been the standard initial treatment, yielding prolonged remissions in over 60% of patients with advanced-stage disease. Several attempts to enhance the outcomes of this regimen over the last two decades have shown limited success. Various novel therapeutic approaches have recently emerged in lymphoma, demonstrating promising results. These include small molecules, novel monoclonal antibodies, antibody–drug conjugates (ADC), bispecific antibodies (BsAbs), and chimeric antigen receptor (CAR) T-cell therapy. This review explores recent advancements in therapeutic strategies for DLBCL and their potential impact on the initial management of DLBCL patients.

## 1. Introduction

Diffuse large B-cell lymphoma (DLBCL) represents the predominant subtype of lymphoma, accounting for approximately 30% of non-Hodgkin’s lymphoma cases. Rather than being a homogeneous disease, DLBCL comprises diverse entities characterized by distinct biology and clinical features [1,2]. Early gene-expression studies identified two patient subgroups based on the cell of origin (COO): germinal center B-cell-like (GCB) and activated B-cell-like (ABC), with some cases remaining unclassified, exhibiting differences in prognosis [3,4,5,6]. The COO is commonly determined in routine practice using immunohistochemistry (IHC) algorithms that catalog patients into the dichotomic subgroups of GCB and non-GCB cases. More recent studies incorporating molecular and genetic data have identified 5–7 molecular subgroups, providing a more accurate characterization of the heterogeneity within this disease [7,8,9,10].

Initial treatment with R-CHOP (rituximab, cyclophosphamide, doxorubicin, vincristine, and prednisone) achieves long remissions in more than 60% of patients with advanced-stage disease. However, 10–15% of patients exhibit refractoriness to this regimen, while 20–25% experience relapse following an initial treatment response, with a majority of relapses occurring within the first 24 months [11,12]. Over the last two decades, several efforts have been made to improve the outcome obtained with this regimen without achieving a significant benefit over R-CHOP. More recently, different novel therapies are being developed in the field of lymphoma with promising results, which have prompted some of these agents to be explored in the initial management of patients with DLBCL, including small molecules targeting specific pathways of lymphoma and, particularly, different immunotherapy strategies with novel monoclonal antibodies, antibody–drug conjugates (ADC), bispecific antibodies (BsAbs), and chimeric antigen receptor (CAR) T-cell therapy.

In this review, we aim to examine recent advances in the therapeutic strategies being developed for DLBCL and their potential implications on the initial management of patients with DLBCL.

## 2. Intensifying Chemotherapy Did Not Yield Improvement over R-CHOP

Several attempts to enhance the treatment outcome in DLBCL have focused on intensifying chemotherapy by either increasing the dose intensity or the treatment density by reducing the interval between treatment cycles.

### 2.1. Intensified Regimens

In this regard, dose-intensified regimens, such as R-megaCHOEP [13], or strategies adding consolidation with autologous stem-cell transplantation [14], have not yielded a survival benefit. One exception to the negative results obtained with this strategy is the LNH03-2B study conducted by the GELA group (Groupe d’Etude des Lymphomes de l’Adulte), which evaluated the intensified regimen of ACVBP plus rituximab (rituximab, doxorubicin, cyclophosphamide, vindesine, bleomycin, and prednisone [R-ACVBP] administered every two weeks for four cycles, followed by consolidation with methotrexate R-ifosfamide, etoposide, and cytarabine) vs. R-CHOP in younger patients (<60 years) with an age-adjusted international prognostic index equal to 1 [15]. In this population of low-risk patients with DLBCL, this intensified combination demonstrated a significant improvement in progression-free survival (PFS) and overall survival (OS) compared to R-CHOP (3-year PFS 87% [95% CI, 81–91] vs. 73% [66–79]; HR 0.48 [0.30–0.76]; *p* = 0.0015, and OS 92% [87–95] vs. 84% [77–89]; HR 0.44 [0.28–0.81]; *p* = 0.0071). However, this regimen was associated with a significant increase in hematologic toxicity. The heightened toxicity, along with the complexity of the treatment and the study’s focus on low-risk patients, has limited the extended use of this regimen.

More recently, a phase III trial comparing the intensified regimen DA-EPOCH-R (dose-adjusted etoposide, prednisone, vincristine, cyclophosphamide, doxorubicin, and rituximab) vs. R-CHOP did not demonstrate a benefit in PFS or OS, whereas DA-EPOCH-R was associated with increased toxicity [16]. Moreover, a large randomized study enhancing the anti-CD20 antibody from rituximab to obinutuzumab also did not yield a benefit in terms of PFS or OS [17].

On the other hand, increasing dose density with R-CHOP every 14 days (R-CHOP-14) instead of 21 (R-CHOP-21) also did not improve patient outcomes [18,19]. Moreover, eight cycles of R-CHOP did not provide a benefit over six cycles of treatment [20]. These studies confirmed six cycles of R-CHOP-21 as the standard of care for the initial treatment of patients with DLBCL.

### 2.2. Escalation of Treatment Based on Interim PET in DLBCL and Maintenance Strategies

Another attempt to improve the results of R-CHOP has involved applying intensified treatment to patients at high risk of treatment failure based on interim PET results. In this context, the PETAL study assessed a treatment escalation strategy using an intensive Burkitt’s lymphoma protocol, consisting of six cycles of an intensive methotrexate and cytarabine-based regimen in PET-positive patients after two cycles of R-CHOP, compared to receiving six additional cycles of standard R-CHOP [21]. The study did not observe a benefit of the intensified arm in terms of event-free survival (EFS) or OS, whereas the Burkitt protocol resulted in significantly more toxicity [21].

Finally, several phase III studies evaluating different maintenance treatment strategies, including rituximab, lenalidomide, enzastaurin, or everolimus, have not improved survival in the context of DLBCL patients [22,23,24,25].

## 3. Molecular Targeted Agents Plus R-CHOP

The two major subgroups of DLBCL according to the COO, GCB, and ABC, identified by gene-expression profiling, differ in the activation of distinctive signaling pathways, with the ABC subgroup relying on chronic B-cell receptor signaling and the constitutive activation of nuclear factor κB (NF-κB) [26]. The development during the last decade of molecular-targeted agents directed to these pathways, including lenalidomide, bortezomib or BTK inhibitors (BTKi) such as ibrutinib, together with encouraging results from preliminary and single-arm phase II studies [27,28,29,30,31,32,33,34], generated significant interest in evaluating strategies based on combining R-CHOP with targeted agents, particularly in the ABC group of patients.

### 3.1. Randomized Trials with Targeted Agents in Front-Line DLBCL

However, different randomized studies assessing the combination of ibrutinib [35], lenalidomide [36], or bortezomib [37,38] with R-CHOP did not demonstrate a clear benefit over R-CHOP alone, failing to confirm the potential advantage of this strategy, even though these studies were directed toward non-GCB patients by IHC [35] or ABC patients identified by GEP (Table 1) In the REMoDL-B, although there was no restriction according to COO, outcomes were analyzed according to COO by GEP, with no clear benefit when the analysis was restricted to the ABC patients [37].

Different factors have been suggested to explain the negative results of these studies, including a potential selection bias for more favorable patients, and treatment tolerance. It should be noted that some of these studies required central testing to confirm a non-CGB or an ABC phenotype, with a prolonged time from diagnosis to treatment (e.g., 27 days in the PHOENIX trial evaluating ibrutinib with R-CHOP [35], and 31 days in the ROBUST study assessing lenalidomide + R-CHOP [36]), potentially limiting the inclusion of more aggressive cases. On the other hand, differences in treatment tolerance could have impacted the benefit obtained from the combinations of R-CHOP plus targeted agents. In the PHOENIX study, older patients (≥60 years) receiving ibrutinib plus R-CHOP experienced increased toxicity, with a higher proportion of patients unable to complete the six cycles of treatment compared to younger patients. A subgroup analysis of this trial focusing on patients younger than 60 years demonstrated that ibrutinib plus R-CHOP improved EFS (HR, 0.579), PFS (HR, 0.556), and OS (HR, 0.330), with a similar proportion of patients completing six cycles of R-CHOP compared to the standard arm with R-CHOP alone [35].

The results of upcoming studies, such as the ESCALADE study (NCT04529772), a phase III trial evaluating the addition of acalabrutinib, more selective BTKi, to R-CHOP in younger patients with DLBCL (<70 years), should contribute to clarifying the benefits of this strategy in this patient population.

### 3.2. Future Directions with Molecular Targeted Agents in DLBCL

Despite the previously reported negative results from these phase III trials, recent data have renovated interest in this strategy. In this regard, a more mature 5-year follow-up of the REMoDL-B study showed that, whereas there was no overall benefit of adding bortezomib on PFS or OS in the overall population of the study, patients with ABC DLBCL experienced improved PFS and OS with bortezomib plus B-CHOP (5-year OS 67% with R-CHOP vs. 80% with RB-CHOP (HR, 0.58; 95% CI, 0.35 to 0.95; *p* = 0.032)). In addition, patients classified as molecular high-grade (MHG) with GEP—a more aggressive subtype characterized by a proliferative phenotype closely related to centroblasts—exhibited higher 5-year PFS: 29% vs. 55% (HR, 0.46; 95% CI, 0.26 to 0.84) with bortezomib plus R-CHOP compared to R-CHOP [40].

Moreover, DLBCL’s biological heterogeneity extends beyond the subgroups derived from the COO, with newly identified molecular subgroups contributing to a better understanding of the pathogenesis of the disease and potentially predicting responses to therapy [7,8,9,10]. A subsequent analysis of younger patients enrolled in the PHOENIX study based on the novel molecular subtypes identified two genetic subtypes of DLBCL (MCD and N1), showing a specific benefit from adding ibrutinib to R-CHOP. In these patients, the 3-year EFS was 100%, whereas it was significantly lower for those treated with R-CHOP alone in the MCD and N1 subtypes (42.9% and 50%, respectively) [42]. Of note, these two molecular subtypes, MCD and N1, highly rely on the BCR and NF-kB pathways, providing a rationale for the particular improved outcomes observed with the addition of ibrutinib. Future clinical trials evaluating targeted molecular agents in DLBCL should ideally incorporate clinical and molecular characterization of patients with DLBCL and prospectively assess the potential of a more tailored strategy.

Finally, the combination of targeted agents may exhibit synergy in DLBCL, particularly in specific subgroups. In this regard, the Smart Start study evaluated the combination of lenalidomide and ibrutinib in the non-GCB subset. This phase II study enrolled 60 patients with newly diagnosed non-CGB DLBCL who received rituximab, lenalidomide, and ibrutinib (RLI) followed by sequential chemotherapy. Two cycles of initial RLI achieved an overall response rate (ORR) of 86.2% [43]. This study suggests the potential for developing molecular-targeted, non-chemotherapy-based regimens for newly diagnosed patients with DLBCL.

## 4. Novel Strategies in the Initial Management of Advance-Stage DLBCL

The introduction of novel monoclonal antibodies, antibody–drug conjugates (ADC), bispecific antibodies (BsAbs), and chimeric antigen receptor (CAR) T-cell therapy is changing the landscape of treatment for lymphoproliferative diseases, particularly for DLBCL patients. The encouraging results that these novel therapies initially obtained in the context of relapse or refractory (R/R) disease are motivating their assessment in the initial management of patients with DLBCL.

### The Phase III POLARIX Study

The first example of the success of this strategy has been the POLARIX study, assessing polatuzumab—an ADC conjugated to the microtubule-disrupting agent monomethyl auristatin E (MMAE) and targeting CD79b—in previously untreated patients with DLBCL. CD79b is a component of the BCR and is ubiquitously expressed on DLBCL cells [44]. Polatuzumab had previously shown activity in DLBCL and is currently approved in the R/R setting in combination with rituximab and bendamustine [45].

The POLARIX study was designed as a double-blind, international phase III trial, combining polatuzumab with R-CHP (excluding vincristine from the regimen to avoid increasing the risk of overlapping neurological toxicity) and comparing this experimental arm to standard R-CHOP in previously untreated patients with DLBCL aged 18–80 years with an IPI of 2–5 [41]. The study demonstrated superior PFS for pola-R-CHP vs. R-CHOP (HR, 0.73; 95% CI, 0.57 to 0.95; *p* < 0.02), with an estimated 2-year PFS rate of 76.7% (95% CI, 72.7 to 80.8) vs. 70.2% (95% CI, 65.8 to 74.6), although this did not translate into a difference in OS. The safety profile was similar for both arms of the study, including rates of grade 3–4 adverse events and peripheral neuropathy. A subgroup analysis suggested a major benefit for adding polatuzumab in male patients over 60 years who had an IPI of 3–5 and an ABC phenotype, although it should be noted that the study was not powered to address specific subgroups.

Of particular interest is the apparent predominant efficacy in ABC cases. This differential activity according to the COO seems consistent in different clinical studies evaluating polatuzumab beyond the POLARIX trial [46,47]. Different factors have been proposed to explain the higher efficacy of polatuzumab in the ABC-type DLBCL, including a potential inhibition of BCR signaling through CD79b internalization by polatuzumab binding, taking into account the greater dependency on BCR in ABC DLBCL. Thus, polatuzumab would exert a dual mechanism on ABC cases, involving microtubule disruption by the payload cytotoxic agent MMAE together with the abrogation of BCR signaling [47].

## 5. Future Directions in the Initial Management of Patients with DLBCL

### 5.1. Novel Monoclonal Antibodies

Beyond polatuzumab, other novel monoclonal antibodies are under evaluation in the front-line setting in DLBCL. Tafasitamab, is an Fc-enhanced monoclonal antibody targeting CD19 antigen. The phase II L-MIND study, combining tafasitamab and lenalidomide, obtained encouraging responses in patients with R/R DLBCL [48], forming the basis for its approval in the relapse setting. These results have led to the assessment of tafasitamab as an initial treatment for patients with DLBCL. The phase Ib First-MIND study demonstrated that the combination of tafasitamab and lenalidomide added to R-CHOP was feasible [49], and this combination is currently being tested in a randomized trial against R-CHOP in the Front-MIND phase III study (Table 1).

### 5.2. CAR-T-Cell Therapy

CAR-T-cell therapy represents a significant advancement in the treatment of R/R DLBCL patients. It was initially approved after at least two prior lines of therapy [50,51,52] and subsequently as a second-line therapy in refractory or early relapse (within 12 months) after first-line chemoimmunotherapy, based on positive results obtained in the ZUMA-7 and TRANSFORM phase III clinical trials [53,54]. Building on these promising data, CAR-T therapy is being explored in earlier lines of therapy. In this regard, the ZUMA-12 trial, a multicenter single-arm phase II study, was the first prospective study to evaluate CAR-T-cell therapy as part of the first-line treatment in high-risk patients with large B-cell lymphoma (LBCL).

In this study, high-risk patients were identified by either the presence of MYC and BCL2 and/or BCL6 rearrangements (double- or triple-hit lymphomas) or high–intermediate- or high-risk IPI scores (≥3), together with an interim PET-positive period after two cycles of chemoimmunotherapy. Forty patients were thus treated with axicabtagene ciloleucel (axi-cel), achieving a complete response rate (CRR) of 78% (95% CI, 62–90) and an ORR of 89% (95% CI, 75–97), with a median EFS and PFS not reached but with a short median follow-up of 15.9 months [55]. These encouraging results have prompted the evaluation of axi-cel in a phase III randomized trial, the ZUMA-23 [56], in high-risk LBCL (defined as IPI 4–5) compared to standard R-CHOP or DA-EPOCH-R chemoimmunotherapy. Patients will receive one cycle of chemoimmunotherapy and then will be randomized to receive axi-cel or continue with standard chemoimmunotherapy. This represents the first phase III trial to assess CAR-T-cell therapy as a front-line treatment in DLBCL (Table 1).

### 5.3. Bispecific Antibodies (BsAbs)

BsAbs are another promising therapy for DLBCL that are a focus of intensive clinical research. The BsAbs glofitamab and epcoritamab, both targeting CD20 and CD3, have been recently approved in the R/R setting based on the efficacy and safety data obtained in phase II studies as single-agent therapy [57,58]. BsAbs are currently being evaluated in combination with R-CHOP or Pola-R-CHP as first-line treatment in DLBCL [59,60,61]. A phase Ib study combining glofitamab plus R-CHOP showed that this combination was feasible in 56 previously untreated patients with DLBCL, with a low incidence of cytokine release syndrome (CRS) (10.7% of any grade CRS, with no severe grade 3–5 CRS events). Importantly, the dose intensity of R-CHOP was maintained in all patients, and the ORR obtained with this combination was 93.5%, including a CRR of 76.1% [59]. Epcoritamab in combination with R-CHOP is also being evaluated in an ongoing phase I/II study in previously untreated high-risk patients (IPI ≥ 3) with DLBCL. In the last reported data cut-off of the study, among 31 efficacy-evaluable patients, the ORR was 100% and the CRR was 77%. Notably, 11 patients with double-hit/triple-hit DLBCL had similar response rates, with an encouraging CRR of 82% (9/11). The median dose intensity for R-CHOP was ≥95%, and CRS was mostly low grade (57% G1–2, 2% G3) [61].

These promising results have supported the evaluation of both BsAbs, glofitamab and epcoritamab, in randomized phase III trials in the front-line treatment of DLBCL patients. Glofitamab in combination with pola-R-CHP is being compared to pola-R-CHP in the phase III trial NCT06047080, whereas the phase III trial EPCORE DLBCL-2 (NCT05578976) is evaluating subcutaneous epcoritamab plus R-CHOP vs. R-CHOP in newly diagnosed DLBCL patients (Table 1).

### 5.4. Immune Checkpoint Blockade

Although immune checkpoint inhibitors (ICIs) blocking the PD-1/PDL-1 axis have exhibited robust activity in other lymphoid malignancies, such as Hodgkin’s lymphoma or primary mediastinal B-cell lymphoma, initial studies evaluating ICIs in DLBCL showed limited efficacy [62,63]. In this sense, the combination of durvalumab (an anti-PDL-1 antibody) in combination with R-CHOP in newly diagnosed high-risk patients with DLBCL in a phase II study did not show a benefit over R-CHOP, with an ORR of 73% and a CRR of 54.1% [64]. Despite these results, the combination of ICIs with other immunotherapy strategies including BsAbs or CAR-T to potentiate the activity of these treatments is being evaluated in different studies. Finally, other strategies, such as those targeting CD47—the anti-phagocytic “do not eat me” signal—have shown preliminary promising results in patients with R/R DLBCL and are another area of active research [65,66]. In this regard, the combination of magrolimab (Hu5F9-G4), an antibody targeting CD47, with rituximab showed an ORR of 40%, including a CRR of 33%, in 15 patients heavily pretreated with R/R DLBCL [66].

## 6. High-Grade B-Cell Lymphoma (HGBCL) with MYC and BCL2 and/or BCL6 Rearrangements

Double-hit lymphoma (DHL) is characterized by the rearrangement of *MYC* and additional rearrangements of *BCL2*, *BCL6*, or both (triple-hit lymphoma). Recently, both in the latest WHO-HAEM5 revision and in the ICC (International Consensus Classification), it has been considered to include only cases with *MYC* and *BCL2* rearrangements within DH lymphomas, with or without additional rearrangements of *BCL6*, thus excluding lymphomas with rearrangements of only *MYC* and *BCL6* [1,2]. In the WHO classification, patients with *MYC* and *BCL6* rearrangements are classified as a subtype of DLBCL or as HGBCL NOS, depending on their morphology, while the ICC has created a new provisional entity for these patients (HBGBL-DH-BCL6). Lymphomas with *MYC* and *BCL2* rearrangements comprise a biologically more homogeneous group both in gene-expression profile (germinal center origin profile) and mutational profile, unlike cases with *MYC* and *BCL6* rearrangements, which include a more heterogeneous variety of cases, including cases with “pseudo” DH where *MYC* rearranges with *BCL6* [1,2,67] Clinical data specifically on DH-BCL6 cases are still limited, and although retrospective studies have reported variable prognosis, recent data suggest a more comparable outcome to DLBCL than to DH-BCL2 lymphoma cases [68].

Standard treatment based on R-CHOP has been associated with poor outcomes in several studies, and therapeutic approaches based on more intensive chemotherapy regimens, such as DA-EPOCH-R, have commonly been used [69,70]. The novel therapies that are being introduced in DLBCL may offer a promising alternative for these patients. Although CAR-T-cell trials have not specifically targeted patients with DH lymphoma, these patients have been represented in these studies. The JULIET trial with tisagenlecleucel included 19 patients with DH lymphoma (20%). Among these patients, the ORR was 50% and the CRR was 25%, similar to that obtained in the general study population [51]. The TRANSCEND NHL 001 trial with lisocabtagene maraleucel included 36 patients with DH lymphoma (13%), with ORR and CRR values also comparable to those of the general trial population [52]. Finally, the ZUMA-1 study (axicabtagene ciloleucel) included 11 patients with DH lymphoma and also demonstrated similar results compared to the non-DH population [71]. Beyond pivotal studies, several retrospective series also suggest that CAR-T-cell therapy could overcome the negative prognostic impact of *MYC* and *BCL2* rearrangement [72,73].

Patients with DH lymphoma have also been included in studies evaluating other novel therapies in DLBCL beyond CAR-T therapy, although the representation of these patients has often been limited. In this regard, the phase II trial of loncastuximab, an anti-CD19 ADC conjugated to a pyrrolobenzodiazepine dimer, included 15 cases of DH, with an overall response rate of 33%, all of them being a CR, with a median response duration of 13 months [74]. Finally, in the pivotal trial of glofitamab in R/R DLBCL, 20 patients (13%) with DH were included with a CR rate of 20% [58].

The ongoing studies exploring these therapies in the front-line setting, including CAR-T-cell therapy or BsAbs, should clarify their potential as initial treatment for this high-risk patient population.

## 7. Elderly and Frail Patients with DLBCL

Older patients with DLBCL are usually treated with attenuated doses of chemotherapy such as R-mini-CHOP, which obtained an ORR of 73% and a CRR of 62% with a 2-year OS of 50% in patients over 80 years of age in a phase II multicentric study [75]. Different studies exploring novel therapies are being conducted specifically targeting this patient population. In this regard, the SENIOR trial evaluated the addition of lenalidomide to R-mini-CHOP in comparison to R-mini-CHOP in patients of 80 years or older, without showing differences in terms of OS between the two arms of the study [39]. Phase III trials evaluating pola-mini-R-CHP (POLAR BEAR Trial, NCT04332822), or the combination of acalabrutinib plus R-mini-CHOP (ARCHED, NCT05820841), over R-mini-CHOP in older patients with DLBCL are currently ongoing.

Finally, the encouraging results obtained with novel therapies offer the opportunity to develop non-chemotherapy-based regimens in frail patient populations with DLBCL. An example of this strategy is the ongoing cohort of the NCT03677154 study, combining the CD20 and CD3 BsAbs mosunetuzumab and polatuzumab in elderly unfit/frail patients with newly diagnosed DLBCL [76].

## 8. Concluding Remarks

During the last two decades, several efforts have been made to improve the outcomes of R-CHOP as a first-line treatment in DLBCL with limited success. However, the recent introduction of different novel therapies is changing the landscape of treatment for patients with DLBCL. The encouraging results achieved by these treatments in the R/R setting have led to the exploration of these therapies in the initial management of DLBCL patients. The POLARIX study, adding the ADC polatuzumab to R-CHP, has been the first example of the success of this strategy, but several ongoing phase III trials evaluating combinations with novel monoclonal antibodies (BsAbs) or exploring CAR-T in the front-line setting should provide a better understanding of the potential of these therapies, including their benefit in high-risk patients such as those with DH lymphoma. On the other hand, studies evaluating targeted molecular agents in DLBCL have not shown a clear benefit in DLBCL and are not yet incorporated into the clinical setting outside the context of clinical trials. However, future trials with a more tailored strategy according to a more detailed molecular characterization of patients with DLBCL should help assess the benefit of these agents and move towards individualized strategies based on both a clinical and molecular characterization of patients.

Finally, these novel therapies open the door to non-chemotherapy-based regimens that could offer novel treatment options for patients not suitable for chemotherapy, including elderly or frail patients.

## Figures and Tables

**Table 1:** Summary of recent and ongoing phase III trials in untreated patients with advanced-stage diffuse large B-cell lymphoma.

Reference	Regimen	N	Patient Population	Outcome	*p*-Value
Schmitz et al. [13] DSHNHL 2002-1 trial	R-CHOEP-14 vs. R-megaCHOEP	275	Age ≤ 60 y; aaIPI = 2–3	3-y EFS 70% vs. 61% 3-y OS 85% vs. 77%	NS NS
Stiff et al. [14]	R-CHOP vs. R-CHOP + ASCT	370	Age ≤ 65 y; aaIPI = 2–3	2-y PFS 55% vs. 69% 2-y OS 71% vs. 74%	0.005 NS
Recher et al. [15] LNH03-2B trial	R-CHOP vs. R-ACVBP	380	Age < 60 y; aaIPI = 1	3-y PFS 73% vs. 87% 3-y OS 84% vs. 92%	0.002 0.007
Bartlett et al. [16] Intergroup Trial Alliance/CALGB 50303	R-CHOP vs. DA-EPOCH-R	491	Age ≥ 18 y	2-y PFS 76% vs. 79% 2-y OS 86% vs. 87%	NS NS
Vitolo et al. [17] GOYA trial	R-CHOP vs. Obinutuzumab-CHOP	1418	aaIPI ≥ 2, or IPI = 1 and age ≤ 60 y, or IPI = 0 and bulky (≥7.5 cm)	3-y PFS 67% vs. 70% 3-y OS 81% vs. 81%	NS NS
Cunningham et al. [18]	R-CHOP-21 vs. R-CHOP-14	1080	Age ≥ 18 y	2-y PFS 75% vs. 75% 2-y OS 81% vs. 83%	NS NS
Delarue et al. [19] LNH03-6B trial	R-CHOP-21 vs. R-CHOP-14	602	Age 60–80 y; aaIPI ≥ 1	3-y EFS 60% vs. 56% 3-y OS 72% vs. 69%	NS NS
Dührsen et al. [21] PETAL trial	R-CHOP-21 vs. Burkitt’s lymphoma protocol	108 (PET+ arm)	Age 60–80 y; PET + after R-CHOPx2	2-y EFS 42% vs. 32% 2-y OS 64% vs. 55%	NS NS
Younes et al. [35] PHOENIX trial	R-CHOP vs. Ibrutinib-R-CHOP	838	Age ≥ 18 y; R-IPI ≥ 1; non-GCB subtype by IHC (Hans algorithm)	3-y EFS 67% vs. 70% 3-y OS 81% vs. 83%	NS NS
Nowakowski et al. [36] ROBUST trial	R-CHOP vs. Lenalidomide-R-CHOP	570	Age ≥ 18 y; IPI ≥ 2; ABC subtype by GEP (NanoString)	2-y PFS 64% vs. 67% 2-y OS 80% vs. 79%	NS NS
Oberic et al. [39] SENIOR trial	R-mini-CHOP vs. Lenalidomide-R-mini-CHOP	249	Age ≥ 80 y	2-y PFS 56% vs. 55% 2-y OS 66% vs. 66%	NS NS
Davies et al. [37,40] REMoDL-B trial	R-CHOP vs. Bortezomib-R-CHOP	918	Age ≥ 18 y	30-m PFS 70% vs. 74% 30-m OS 82% vs. 83% *ABC by GEP subgroup:* 5-y PFS 54% vs. 69% 5-y OS 67% vs. 80% *MHG by GEP subgroup:* 5-y PFS 29% vs. 55% 5-y OS 48% vs. 60%	NS NS 0.041 0.032 0.011 NS
ESCALADE study (ACE-LY-312; NCT04529772)	R-CHOP vs. Acalabrutinib-R-CHOP	600 (estimated)	Age ≤ 75 years; IPI ≥1; ABC or unclassified subtypes by GEP	Ongoing	
Tilly et al. [41] POLARIX trial	R-CHOP vs. Pola-R-CHP	879	Age 18–80; IPI 2–5	2-y PFS 70% vs. 77% 2-y OS 89% vs. 89%	0.02 NS
frontMIND study (NCT04824092)	R-CHOP vs. Tafasitamab + lenalidomide + R-CHOP	880 (estimated)	Age 18–80; IPI 3–5; (aaIPI 2–3 if ≤60 years)	Ongoing	
ZUMA-23 study (NCT05605899)	R-CHOP or DA-EPOCH-R vs. Axi-cel	300 (estimated)	Age ≥ 18 y; IPI ≥ 4	Ongoing	
SKYGLO study (NCT06047080)	Pola-R-CHP vs. Glofitamab + Pola-R-CHP	1130 (estimated)	Age 18–80; IPI 2–5	Ongoing	
EPCORE DLBCL-2 (NCT05578976)	R-CHOP vs. Epcoritamab + R-CHOP	900 (estimated)	Age 18–80; IPI 2–5	Ongoing	
POLAR BEAR (NCT04332822)	R-mini-CHOP vs. Pola-R-mini-CHP	300 (estimated)	Age > 80 y or 75–80 y and frail	Ongoing	
ARCHED (NCT05820841)	R-mini-CHOP vs. Acalabrutinib-R-mini-CHOP	330 (estimated)	>80 y or >60 y and ineligible for full-dose R-CHOP	Ongoing	

aaIPI, age-adjusted International Prognostic Index; R-IPI, revised-International Prognostic Index; PFS, progression-free survival; EFS, event-free survival; OS, overall survival; IHC, immunohistochemistry; GEP, gene-expression profiling.

## Data Availability

No applicable.
